# Comparison of Recurrence Risk Prediction by the CanAssist Breast Test and the PREDICT Online Tool in Early Breast Cancer Patients

**DOI:** 10.7759/cureus.77356

**Published:** 2025-01-12

**Authors:** Rajeev Kumar, Garima Daga, Prerit Sharma, Anupam Lahiri, Arnab Chakraborty, Anuj Mehta, DC Doval, Pankaj Goel, Vineet Talwar

**Affiliations:** 1 Surgical Oncology, Breast Services, Rajiv Gandhi Cancer Institute and Research Centre, New Delhi, IND; 2 Surgical Oncology, Rajiv Gandhi Super Speciality Hospital, New Delhi, IND; 3 Surgical Oncology, Rajiv Gandhi Cancer Institute and Research Centre, New Delhi, IND; 4 Medical Oncology, Rajiv Gandhi Cancer Institute and Research Centre, New Delhi, IND; 5 Medical Oncology, Breast Services, Rajiv Gandhi Cancer Institute and Research Centre, New Delhi, IND

**Keywords:** canassist breast, early breast cancer, estrogen & progesterone receptor, her2/neu negative, hormone receptor positive, kappa correlation coefficient., online prognostic tools, predict

## Abstract

Introduction

Several methods, such as multi-marker (both gene/protein) tests and online/free tools, are available for the prognostication of early breast cancer (EBC) patients. This article compares the risk assessment between the immunohistochemistry-based (IHC) CanAssist Breast (CAB) test and the online/free prognostic tool PREDICT in EBC patients treated at Rajiv Gandhi Cancer Institute and Research Centre (RGCIRC) between May 2017 and June 2022.

Methodology

The current study cohort comprises 130 patients. Risk proportions were assessed by CAB and the online/free tool PREDICT. Concordance between the risk groups of CAB and PREDICT was assessed by the kappa coefficient, which was used to evaluate the significance.

Results

The low-risk (LR) and high-risk (HR) proportions for CAB and PREDICT were 61:39 and 46:54 percent, respectively. CAB stratified a significantly (*P*=0.027) higher number of patients as LR compared to PREDICT. Interestingly, in the subgroup analysis of the age and clinicopathological parameters CAB stratified 28% of patients with grade 3 tumors as LR, whereas PREDICT stratified all grade 3 tumors as HR. The overall treatment compliance using CAB was 89%.

Conclusion

CAB, a relatively new multi-marker prognostic test entailing five relevant protein biomarkers provides augmented and relevant prognostic information over PREDICT in all patients. Thus, CAB helps optimize treatment for HR+/HER2− EBC patients as its risk stratification is independent of age and clinical parameters and assigns recurrence risk based on tumor biology.

## Introduction

Breast cancer is the most diagnosed cancer among women, with an estimated rate of 2.3 million new cases in 2020 accounting for 11.7% of all cancers with a mortality rate of 6.9% in women [1]. By 2040, the projected number of newly diagnosed breast cancers is estimated to expand by over 40% [2]. The clinical outcomes of patients differ between individuals and are largely dependent on the individual clinical parameters and aggressiveness of the tumor based on underlying tumor biology. Assessing the aggressiveness of breast tumors will help clinicians make superior decisions regarding medical treatment planning thereby helping in the prevention of excessive treatment, and thus in turn reducing economic costs [3].

A surge in the availability of medical data along with the application of artificial intelligence approaches has led to the construction of survival prediction models in the field of cancer. Traditional prediction models based on prior knowledge often consider the relationship between dependent variables; in contrast, AI/ML has the potential to learn data models automatically [4]. There are several free web-based prognostic calculators to predict the survival of early breast cancer and PREDICT is one such tool. PREDICT (https://breast.predict.nhs.uk), is an online free-prognostic tool that was established by the United Kingdom in 2010 [5]. The tool includes inputs like age, menopausal status, ER, HER2, Ki67 status, invasive tumor size, tumor grade, number of nodes, mode of detection, type of hormonal therapy (HT)/chemotherapy (CT) and provides 5- and 10-year survival estimates as well as treatment benefit predictions [6]. There have been a small number of studies to confirm the prognostic ability of these programs in Asian populations [7,8]. Despite the widespread use of PREDICT, data comparing the validity are limited and controversial [9,10]. Conversely, CanAssist Breast (CAB) is a prognostic test that has been developed on Asian patients by integrating multiple markers using an IHC approach. It uses a machine learning algorithm to compute a risk score for distant recurrence over a timeline of five years with the inputs from IHC gradings of five biomarkers (CD44, ABCC4, ABCC11, N-Cadherin, and pan-Cadherin) along with three clinical parameters (node status, tumor size, and tumor grade) [11-13]. CAB has been extensively validated in the Southeast Asian breast cancer population [14] including the population of different races/ethnicities around the globe, especially from Europe and the USA [15], making it more suitable for the global population, unlike PREDICT.

This article aims to compare the risk stratification of the IHC-based Indian test, CAB, and the UK-based free-online tool, PREDICT, systematically and comprehensively in the early-stage breast cancer patients of Rajiv Gandhi Cancer Institute and Research Centre (RGCIRC), New Delhi, India who used CAB to plan adjuvant chemotherapy.

## Materials and methods

Cohort description

This study involved the analysis of 130 tumor specimens of HR-positive, HER2-negative EBC patients from RGCIRC, New Delhi, India, over a period of five years (from May 2017 to June 2022). All the information about the patients and treatment (such as age, year of diagnosis, clinical parameters, and hormone receptor status) was obtained from the histopathology and IHC reports, and treatment follow-up received by the patient was obtained either from the patient or the treating clinician.

Immunohistochemistry and CAB-based risk categorization

The inclusion and exclusion criteria for performing CAB are described previously [11]. CAB was performed on formalin-fixed paraffin-embedded (FFPE) blocks. Five consecutive sections of 3-micron thickness were used for IHC staining of the CAB’s five biomarkers. All the IHC staining was performed at OncoStem's College of American Pathologists (CAP)-certified and ISO 15189-accredited central laboratory. Briefly, IHCs for CAB protein biomarkers were performed as described earlier on an automated Ventana IHC machine [11,12]. This IHC information, along with tumor size, grade, and node status, was used to arrive at a CAB risk score that ranges between 0 and 100 using the CAB algorithm. A cut-off of 15.5 was used to stratify the patients into LR (≤15.5) and HR (>15.5) categories for distant recurrence [13,14].

PREDICT

PREDICT is an online prognostic tool that provides estimates of 5 and 10-year survival with each modality of treatment [5,6]. The PREDICT algorithm includes clinical parameters, menopausal status, and other parameters like mode of cancer detection, HER2 status, as well as Ki-67 status. The current study comprises the Indian population with cut-off ages for the pre and postmenopausal states being ≤ 46 and > 46 years, respectively [16,17]. Further, the Ki67 and mode of detection were selected unknown for all the patients since 95% of this cohort did not have the information on the same. Further, for the purpose of analysis, we have considered hormone therapy (HT) for five years followed by third-generation chemotherapy (CT) along with five years of overall survival rate. Chemotherapy benefit difference between third-generation CT and HT resulting < 2% is considered LR.

Ethical statement

The current report involves an analysis of existing patient data that were captured during regular procedures as part of routine patient care. No additional tissue-based assessments were performed. EC approval was obtained from the Rajeev Gandhi Cancer Institute and Research Centre’s Institutional Review Board with approval number RGCIRC/IRB-BHR/72/2024.

Statistical analyses

The comparison of risk proportions and the kappa correlation coefficient between the risk groups of CAB and PREDICT was computed by MedCalc software [18]. The chi-squared test on MedCalc was used to calculate the P values. A P value of < 0.05 is considered statistically significant.

## Results

Cohort description/patient characteristics

This study cohort consisted of a total of 130 HR+, HER2- breast EBC patients. Patients aged ≤48 years were 18% and >48 years were 82% (median age 60 years; range: 35 to 81 years). Seventy-five percent of the cohort had T2 (2.1-5cm) tumors followed by 25% with T1 (0-2cm) tumors (median T size 2.5 cm; range: 0.8 to 5.0 cm). 74% of the cohort had a node-negative disease and 26% were N1 tumors. Fifty-five percent of the patients had G2 tumors followed by 28% G3 and 17% G1 tumors (Table 1).

**Table 1:** Patient demographics of the study cohort

Clinicopathological features	Subgroups	n	%
	total	130	100
Age (years)	≤ 48	24	18
	> 48	106	82
	Median	60 (35 to 81)	
Tumor (T) size (cm)	T1 (0-2cm)	33	25
	T2 (2.1-5cm)	97	75
	Median	2.5 (0.8 to 5.0)	
Node (N) status	N0	96	74
	N1 (1-3 nodes)	34	26
Tumor grade (G)	G1	22	17
	G2	72	55
	G3	36	28

Risk proportions by CAB and PREDICT

**Figure 1:**
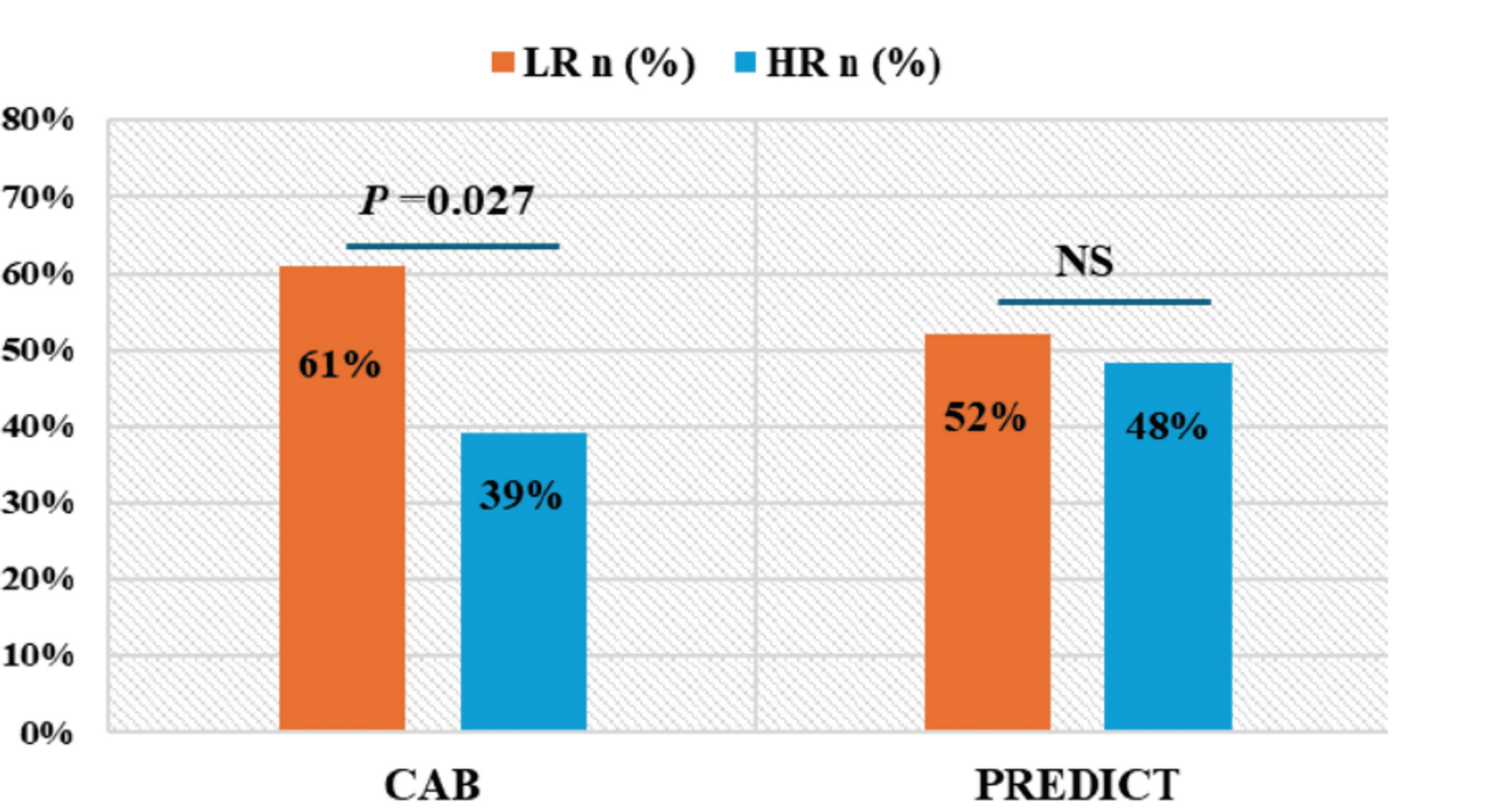
Risk proportions by CAB and PREDICT The chi-squared test on MedCalc was used to calculate the p-value. CAB: CanAssist Breast, LR: Low Risk, HR: High Risk, NS: Not Significant

CAB significantly stratified (P=0.027) a higher number of patients as LR with 61% (n=79) and 39% (n=51) of patients as HR, whereas PREDICT stratified 52% (n=67) of the patients as LR and 48% (n=63) of patients as HR (P=0.689) (Figure 1). The bar graph represents the significant difference in risk proportions by CAB and PREDICT.

LR patients by CAB vs PREDICT across various clinical subgroups

Further, we compared LR patients of CAB and PREDICT across all the clinical subgroups. CAB identified 58% of patients aged ≤48 years as LR while by PREDICT, they were 50%. For patients aged above 48 years, LR percentages were higher by CAB (61%) than by PREDICT (52%). CAB stratified a greater number of the patients with T1 tumors (91%), T2 tumors (51%), N0 (74%), and N1 (24%) as LR when compared to the LR of PREDICT (T1: 81%, T2: 41%, N0: 64% & N1: 18%). All patients belonging to the G1 subgroup came under the PREDICT-LR category while 95% were stratified as LR by CAB. In the G3 subgroup, CAB stratified 28% as LR while for PREDICT, none of the patients were LR (Table 2).

**Table 2:** Low-risk proportions of CAB vs PREDICT CAB-CanAssist Breast, LR-Low Risk, HR-High Risk

Clinical Parameters	CAB-LR n (%)	PREDICT-LR n (%)
Age ≤ 48	14 (58%)	12 (50%)
Age > 48	65 (61%)	55 (52%)
T1	30 (91%)	27 (81%)
T2	49 (51%)	40 (41%)
N0	71 (74%)	61 (64%)
N1	8 (24%)	6 (18%)
G1	21 (95%)	22 (100%)
G2	48 (76%)	45 (63%)
G3	10 (28%)	0

Re-stratification of recurrence risk groups by CAB and PREDICT

**Figure 2:**
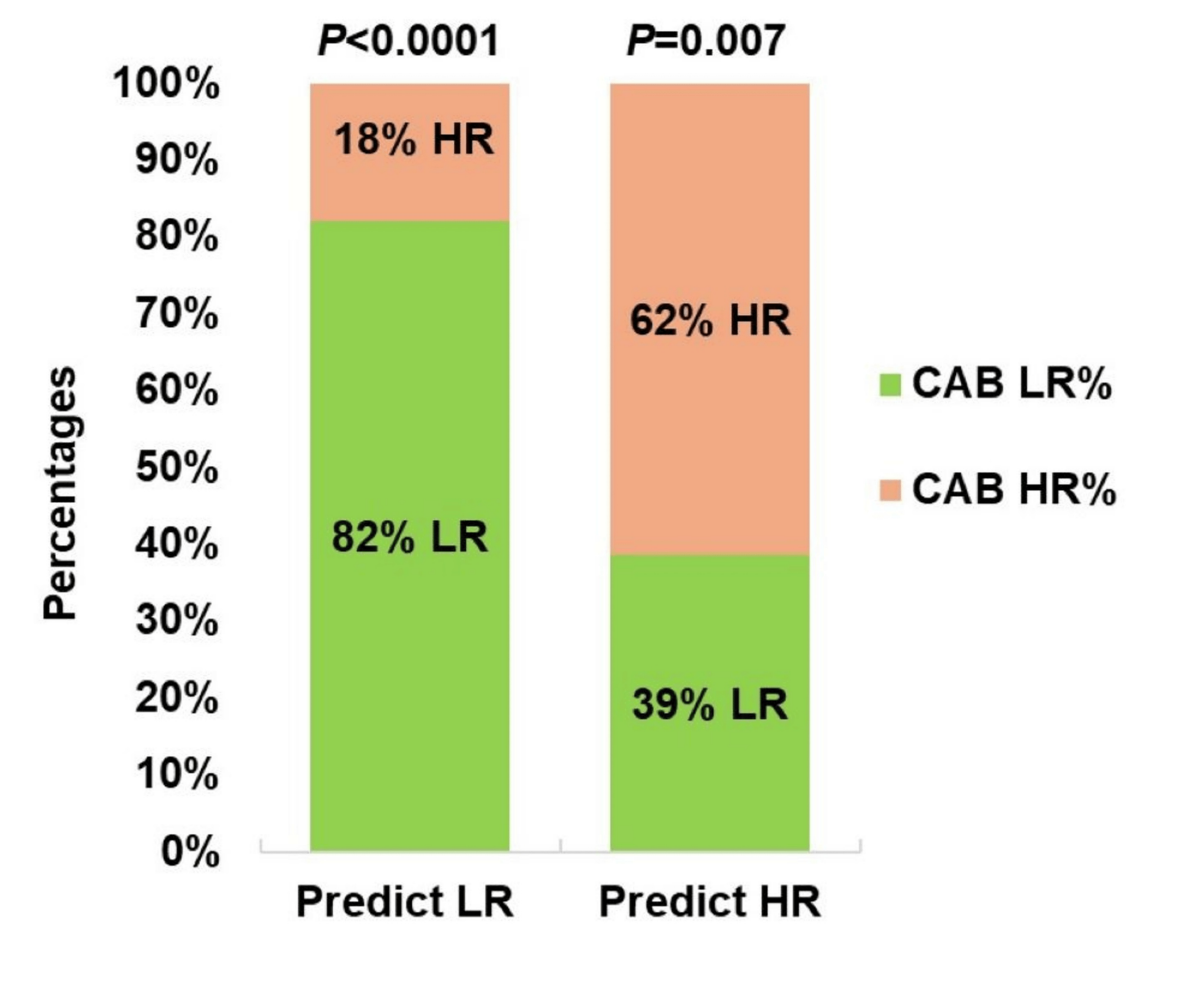
Re-stratification of risk by CAB and PREDICT The bar diagram represents low-risk and high-risk percentages by CAB in PREDICT low-risk and high-risk categories.* *The* *chi-squared test on MedCalc was used to calculate the P value. CAB: CanAssist Breast, LR: Low Risk, HR: High Risk

A re-stratification analysis of the recurrence risk revealed that out of 67 patients from PREDICT-LR, 55 patients were CAB-LR, constituting 82% (95% CI, 48.7 to 74.4), and the rest 12 patients of PREDICT-LR were CAB-HR, constituting 18% (P<0.0001), whereas, out of 63 patients from PREDICT-HR, 24 patients were CAB-LR, constituting 38% (95% CI, 6.5 to 39.5), and the rest 39 patients of PREDICT-HR were also CAB-HR, constituting 62% (P=0.007) (Figure 2).

Concordance between PREDICT and CAB

Between PREDICT and CAB, the concordance was 82% in the LR category, whereas 62% of concordance was observed in the HR category and the overall concordance was 72% (k: 0.442; 95% CI: 0.290 to 0.594), representing a moderate agreement. Across both the age groups, we have observed good agreement (≤ 48 years: 83% (k: 0.666; 95% CI: 0.372 to 0.960).

In the >48 years group, the agreement was only fair (70%; (k: 0.391; 95% CI: 0.218 to 0.563)) between CAB and PREDICT. Interestingly, in clinical subgroups like T1, 96% concordance was observed in the LR category and 33% in the HR category with an overall fair agreement (overall concordance-85%, k: 0.367; 95% CI: -0.05 to 0.794). In the T2 subgroup, overall concordance was 68% with a fair agreement (k: 0.361; 95% CI: 0.179 to 0.544). In the N0 subgroup, these concordances were 87% (LR) and 49% (HR) with an overall fair agreement (overall concordance of 73% (k: 0.377; 95% CI: 0.185 to 0.569). The N1 subgroup shows a poor agreement (k: 0.105; 95% CI: -0.254 to 0.464) and an overall concordance of 71%. Only the G2 subgroup showed any correlation of k: 0.242; 95% CI: -0.012 to 0.472 and a fair agreement (Table 3).

**Table 3:** Concordance and kappa (K) correlation between CAB and PREDICT CAB: CanAssist Breast, LR: Low Risk, HR: High Risk, CI: Confidence Interval, NA: Not Applicable

Parameters	% Concordance in LR between PREDICT and CAB	% Concordance in HR between PREDICT and CAB	Overall concordance (%)	Kappa (K) correlation	95% CI	Agreement
							Total cohort	82	62	72	0.44246	0.29048 to 0.59443	Moderate
Age ≤ 48	92	75	83	0.66667	0.37263 to 0.96070	Good
Age > 48	80	59	70	0.39102	0.21832 to 0.56373	Fair
T1	96	33	85	0.36782	-0.058987 to 0.79462	Fair
T2	73	65	68	0.36198	0.17982 to 0.54413	Fair
N0	87	49	73	0.37756	0.18547 to 0.56964	Fair
N1	33	79	71	0.10526	-0.25414 to 0.46467	Poor
G1	95	0	95	NA	NA	NA
G2	76	48	65	0.24242	0.012346 to 0.47250	Fair
G3	0	72	72	NA	NA	NA

Treatment compliance

Of the 130 patients, treatment follow-up was available for 128 patients. Of these, 79 patients were CAB-LR and 49 patients were CAB HR. This data indicates 97% compliance with respect to CAB LR patients and 76% compliance with respect to CAB HR patients with an overall compliance of 89% to CAB recommendations (Table 4).

**Table 4:** Compliance with CAB recommendations CAB: CanAssist Breast, LR: Low Risk, HR: High Risk

Risk category	Treatment compliance		
	CT (n)	No CT (n)	% of compliance
CAB LR (n=79)	2	77	97
CAB HR (n=49)	37	12	76
Total compliance (%)	89		

## Discussion

Both prognostic and predictive markers are of high relevance in planning a treatment protocol to perceive the finest clinical outcomes for each patient. Adjuvant chemotherapy has been shown to offer protection against distant recurrences in patients with early-stage HR+/HER2- breast cancer but only in a limited number of patients [19]. Therefore, the identification of patients with high-risk disease who could be treated with chemotherapy becomes critical [20]. Clinicians assign risk categories to HR+/HER2- EBC patients based on individual prognostic factors, free online prognostic tools, or expensive multi-marker prognostic tests or tools, thereby identifying high-risk patients [21]. Nottingham Prognostic Index (NPI), modified Adjuvant Online (mAOL), and PREDICT are a few free prognostic tools that clinicians across the globe often use. However, neither multigene nor free tests are developed and validated enough on the Asian breast cancer population, more specifically on Indian breast cancer patients. The effectiveness of a prognostic tool/test for a cohort is well-proven by its cohort-specific validation. In the current report, we show the advantages of CAB, an Indian-based test that incorporates tumor biology parameters over the UK-based PREDICT that integrates proliferation markers for recurrence risk prediction in a hospital-specific cohort from RGCICR.

Cancer recurrence is no longer synonymous with clinical high-risk such as bigger tumors, higher grade or node positivity, or high proliferative marker (Ki67). Underlying complex tumor biology is of paramount importance and gives a true assessment of recurrence risk [22]. It is worth noting that CAB assigns low risk to a significantly greater number of patients as compared to PREDICT, which uses clinical parameters and proliferation markers like Ki67 for assessing chemotherapy benefits to each patient. However, for the purpose of this current study, Ki67 is selected as unknown in the PREDICT tool for all patients due to the absence of Ki67 data for 95% of the cohort. This is due to the lack of a standardized procedure, inter-observer variability, and clinician’s preference for the use of Ki67 [23]. These findings are in line with earlier findings obtained from larger retrospective cohorts [24] and other hospital-based registries [25]. Across clinically high-risk subgroups of patients (T2/G3), CAB showed a higher number of low-risk patients compared to PREDICT, indicating over-treatment can be avoided in these patients. Contrariwise, in the clinically low-risk subgroup (G1), all patients are stratified as low-risk by PREDICT while CAB pointed out 5% as high-risk patients, who may benefit from additional therapies, echoing the value of understanding underlying tumor biology by integrating tumor biomarkers (such as CD44, ABCC4, ABCC11, N-cadherin and pan-cadherin - the five CAB biomarkers) along with clinical parameters. CAB biomarkers represent the aggressive tumor biology and hence can find low-risk patients from clinically high risk and vice versa.

Even though PREDICT has been extensively validated, little is known about their prognostic performance in EBC patients from non-Western, low, or middle-income countries [26,27]. The validation studies conducted in non-European (Asian) countries as well as independent investigations in Western Europe have shown that for several patient categories, such as young women, prognostic calculations of these tools have discrepancies with the observed survival data [28,29]. Unlike PREDICT, CAB is a multi-marker IHC-based prognostic test that considers both tumor biology as well as clinical features for the prediction of risk of recurrence and does not depend on the expression of ER, PR, and HER2. To date, CAB has been extensively validated in multi-ethnic and multi-racial cohorts and was found that CAB’s risk stratification is similar across these diverse BC populations [30]. Further, CAB risk predictions are shown to be valid until 10 years from disease diagnosis in a DUTCH sub-cohort of patients who participated in a prospective trial, TEAM [31]. To date, CAB has been used by more than 350 oncologists across India and the Middle East on ~3500 patients to plan therapy.

The limitation of the study is that the data is constrained by the lack of five-year clinical outcomes for all the patients. In the coming days, we, as clinicians, will keep track of all these patients and compare CAB and PREDICT with respect to clinical outcomes.

## Conclusions

Physicians need reliable tools to assess the prognosis of breast cancer patients and thereby plan effective treatment without compromising on the outcomes. CAB, a test that has been developed on Indian EBC patients and validated on global EBC populations identified a greater number of patients (across all clinical sub-groups) as LR thereby avoiding chemotherapy in these many EBC patients as compared to PREDICT.

## References

[1] Sung H, Ferlay J, Siegel RL, Laversanne M, Soerjomataram I, Jemal A, Bray F (2021). Global cancer statistics 2020: GLOBOCAN estimates of incidence and mortality worldwide for 36 cancers in 185 countries. CA Cancer J Clin.

[2] Arnold M, Morgan E, Rumgay H (2022). Current and future burden of breast cancer: global statistics for 2020 and 2040. Breast.

[3] Li J, Zhou Z, Dong J, Fu Y, Li Y, Luan Z, Peng X (2021). Predicting breast cancer 5-year survival using machine learning: a systematic review. PLoS One.

[4] Obermeyer Z, Emanuel EJ (2016). Predicting the future - big data, machine learning, and clinical medicine. N Engl J Med.

[5] Wishart GC, Azzato EM, Greenberg DC (2010). PREDICT: a new UK prognostic model that predicts survival following surgery for invasive breast cancer. Breast Cancer Res.

[6] Wishart GC, Azzato EM, Greenberg DC (2010). Erratum to: PREDICT: a new UK prognostic model that predicts survival following surgery for invasive breast cancer. Breast Cancer Res.

[7] Phung MT, Tin Tin S, Elwood JM (2019). Prognostic models for breast cancer: a systematic review. BMC Cancer.

[8] Wong HS, Subramaniam S, Alias Z (2015). The predictive accuracy of PREDICT. A personalized decision-making tool for Southeast Asian women with breast cancer. Medicine (Baltimore).

[9] Miao H, Hartman M, Verkooijen HM (2016). Validation of the CancerMath prognostic tool for breast cancer in Southeast Asia. BMC Cancer.

[10] Polchai N, Sa-Nguanraksa D, Numprasit W, Thumrongtaradol T, O-Charoenrat E, O-Charoenrat P (2020). A comparison between the online prediction models CancerMath and PREDICT as prognostic tools in Thai breast cancer patients. Cancer Manag Res.

[11] Ramkumar C, Buturovic L, Malpani S (2018). Development of a novel proteomic risk-classifier for prognostication of patients with early-stage hormone receptor-positive breast cancer. Biomark Insights.

[12] Serkad CP, Attuluri AK, Basavaraj C (2021). Validation of CanAssist Breast immunohistochemistry biomarkers on an automated platform and its applicability in tissue microarray. Int J Clin Exp Pathol.

[13] Attuluri AK, Serkad CP, Gunda A (2019). Analytical validation of CanAssist-Breast: an immunohistochemistry based prognostic test for hormone receptor positive breast cancer patients. BMC Cancer.

[14] Bakre MM, Ramkumar C, Attuluri AK (2019). Clinical validation of an immunohistochemistry-based CanAssist-Breast test for distant recurrence prediction in hormone receptor-positive breast cancer patients. Cancer Med.

[15] Gunda A, Basavaraj C, Serkad V CP (2022). A retrospective validation of CanAssist Breast in European early-stage breast cancer patient cohort. Breast.

[16] Ahuja M (2016). Age of menopause and determinants of menopause age: a PAN India survey by IMS. J Midlife Health.

[17] Ali KY, Erkok U, Mohamed NA, Hilowle NM, Elmi HA, Mohamud RY (2023). Age at natural menopause and influencing factors in women attending the gynecological outpatient clinic at a tertiary care hospital. Int J Womens Health.

[18] (2025). MedCalc statistical software. MedCalc Statistical Software.

[19] Yaghi M, Bilani N, Dominguez B (2023). Efficacy of chemotherapy in patients with HR+/HER2-invasive lobular breast cancer. Cancer Treat Res Commun.

[20] Fasching PA, Kreipe H, Del Mastro L (2024). Identification of patients with early HR+ HER2- breast cancer at high risk of recurrence. Geburtshilfe Frauenheilkd.

[21] Curigliano G, Dent R, Llombart-Cussac A, Pegram M, Pusztai L, Turner N, Viale G (2023). Incorporating clinicopathological and molecular risk prediction tools to improve outcomes in early HR+/HER2- breast cancer. NPJ Breast Cancer.

[22] Hudis CA (2015). Biology before anatomy in early breast cancer - precisely the point. N Engl J Med.

[23] Louis DM, Nair LM, Vallonthaiel AG, Narmadha MP, Vijaykumar DK (2023). Ki 67: A promising prognostic marker in early breast cancer—a review article. Indian J Surg Oncol.

[24] Gunda A, Eshwaraiah MS, Gangappa K, Kaur T, Bakre MM (2022). A comparative analysis of recurrence risk predictions in ER+/HER2- early breast cancer using NHS Nottingham Prognostic Index, PREDICT, and CanAssist Breast. Breast Cancer Res Treat.

[25] Chandra Doval D, Mehta A, Somashekhar SP (2021). The usefulness of CanAssist breast in the assessment of recurrence risk in patients of ethnic Indian origin. Breast.

[26] Leong SP, Shen ZZ, Liu TJ (2010). Is breast cancer the same disease in Asian and Western countries?. World J Surg.

[27] Lao C, Lawrenson R, Edwards M, Campbell I (2019). Treatment and survival of Asian women diagnosed with breast cancer in New Zealand. Breast Cancer Res Treat.

[28] Farooq S, Coleman MP (2005). Breast cancer survival in South Asian women in England and Wales. J Epidemiol Community Health.

[29] Iqbal J, Ginsburg O, Rochon PA, Sun P, Narod SA (2015). Incorrect description for basis of household income. JAMA.

[30] Sengupta AK, Gunda A, Malpani S, Serkad CP, Basavaraj C, Bapat A, Bakre MM (2020). Comparison of breast cancer prognostic tests CanAssist Breast and Oncotype DX. Cancer Med.

[31] Zhang X, Gunda A, Kranenbarg EM (2023). Ten-year distant-recurrence risk prediction in breast cancer by CanAssist Breast (CAB) in Dutch sub-cohort of the randomized TEAM trial. Breast Cancer Res.

